# Characteristics of the Water- and Alkali-Soluble Hemicelluloses Fractionated by Sequential Acidification and Graded-Ethanol from Sweet Maize Stems

**DOI:** 10.3390/molecules24010212

**Published:** 2019-01-08

**Authors:** Xiaopeng Peng, Shuangxi Nie, Xiaoping Li, Xiong Huang, Quanzi Li

**Affiliations:** 1 State Key Laboratory of Tree Genetics and Breeding, Chinese Academy of Forestry, Beijing 100091, China; huangxiong0839@163.com; 2 Guangxi Key Laboratory of Clean Pulp & Papermaking and Pollution Control, Nanning 530004, China; cppkl@gxu.edu.cn; 3 Research Institute of Forestry, Chinese Academy of Forestry, Beijing 100091, China; 4 Jiangsu Key Laboratory for Poplar Germplasm Innovation and Variety Improvement, Nanjing Forestry University, Nanjing 210037, China; xpli@njfu.edu.cn

**Keywords:** sweet maize stems, hemicelluloses, structural features, graded ethanol precipitation, fractionation

## Abstract

Sweet maize stems were treated with hot water and potassium hydroxide to fractionate hemicellulosic polymers. The results showed that the water-soluble hemicelluloses were mainly composed of glucose (27.83%), xylose (27.32%), and galactose (16.81%). In comparison, alkali-soluble hemicelluloses fractionated by acidification and a graded ethanol solution (10%, 20%, 35%, 50%, 65%, and 80%) were mainly composed of xylose (69.73 to 88.62%) and arabinose (5.41 to 16.20%). More highly branched hemicelluloses tended to be precipitated in a higher concentration of ethanol solution, as revealed by the decreasing xylose to arabinose ratio from 16.43 to 4.21. Structural characterizations indicated that alkali-soluble hemicelluloses fractionated from sweet maize stems were mainly arabinoxylans. The results provided fundamental information on hemicelluloses composition and structure and their potential utilization in the fields of biofuels, biochemicals, and biomaterials.

## 1. Introduction

Since excessive consumption of fossil fuels has caused increasing energy and environmental issues, the utilization of renewable, abundant, and inexpensive non-food lignocellulosic biomass is thought to be an effective way to relieve these problems [1]. Lignocellulose is mainly comprised of cellulose (38–50%), hemicelluloses (23–32%) and lignin (15–25%), as well as small amounts of extractives and ash [2]. These components are strongly intermeshed and bonded through non-covalent forces and covalent cross-linkages, forming a lignocellulosic matrix. Hemicellulosic polymer can be converted into chemicals, such as xylitol, lactic acid, furfural, or erythritol [3]. They can also be used as intermediates for hemicellulose-based materials in hydrogels, film, pharmaceuticals, coatings, or papermaking [4,5].

Hemicelluloses are composed of various pentose and hexose units, which are arranged in different proportions and substituents [6,7]. Hemicelluloses are branched heteropolymers with a degree of polymerization around 80 to 200 [8]. The content and chemical structure of hemicelluloses can vary within different biomass types and even in the same plant [9,10]. However, hemicelluloses are usually closely associated with other cell wall components, such as cellulose, lignin, and proteins. The utilization of hemicelluloses is based on their effective fractionation from plant cell walls. Many techniques, including alkali and acid extractions, steam explosion, and hot water extraction, are used to fractionate hemicelluloses from lignocellulose [9]. In these techniques, alkali extraction is well known to disrupt the cell walls and cleave the bonds between hemicelluloses and other components, resulting in the liberation of hemicelluloses in aqueous media [11,12]. Hemicelluloses in alkaline solvent can be separated using acidification precipitation, ethanol precipitation, membrane fractionation, or chromatograph fractionation [9,13,14,15]. Among these methods, both acidification and ethanol precipitation are the most commonly used to separate hemicelluloses from aqueous solvents, because they are both easily conducted and highly efficient. However, separation conditions may affect the composition and yield of hemicelluloses. To our knowledge, the composition, structure, and yield of hemicelluloses precipitated by acidification and graded ethanol solvents from sweet maize have rarely been studied.

In the present study, the composition, structure, and yield of alkali-soluble hemicelluloses from sweet maize stems precipitated by acidification and graded ethanol solvents (10%, 20%, 35%, 50%, 65%, and 80%) were comparatively analyzed. The hemicellulosic fractions were characterized by a high-performance anion exchange chromatography (HPAEC), gel permeation chromatography (GPC), and Fourier Transform Infrared (FT-IR) together with ^1^H and ^13^C NMR spectroscopies.

## 2. Materials and Methods

### 2.1. Materials

Sweet maize stems were obtained from the State Key Laboratory of Tree Genetics and Breeding, Research Institute of Forestry, Chinese Academy of Forestry, China. Air-dried stems were ground and selected to be within the 40 to 80 mesh for subsequent experiments. After drying in an oven at 60 °C for 12 h, the stems were extracted with toluene/ethanol (2:1, *v*/*v*) in a Soxhlet apparatus for 6 h to remove wax and dried at 60 °C for 12 h before use. The chemical composition (weight%) was determined to be 38.1% cellulose, 28.0% hemicelluloses, and 15.0% acid-insoluble lignin, according to the National Renewable Energy Laboratory (NREL) standard analytical procedure. The derivations of these values from their respective means were all <6%. All chemicals were of analytical or reagent grade and used directly without further purification.

### 2.2. Extraction of Hemicelluloses

**Figure 1:**
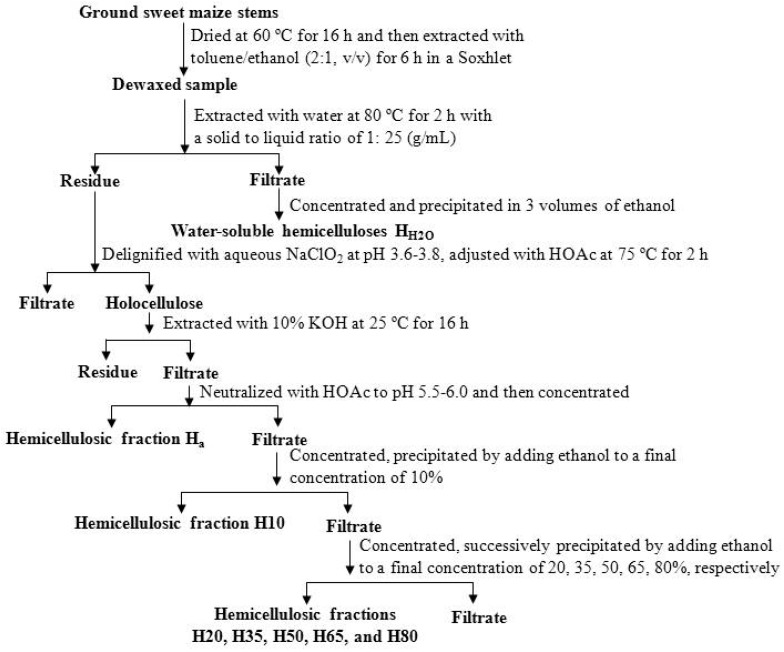
Scheme of hemicellulose fractionation from sweet maize stems.

The dewaxed stems (15.00 g) were firstly extracted with distilled water at 80 °C for 2 h with a solid to liquid ratio of 1:25 g/L. The water-extractable solution was concentrated to about 30 mL and then poured into 90 mL of 95% ethanol while vigorously stirring. The precipitate was centrifuged, freeze-dried, and labeled as H_H2O_. Subsequently, the water-extracted residue was dried at 60 °C for 12 h and delignified using NaClO_2_ at pH 3.6 to 4.0 and adjusted with acetic acid (HOAc) at 75 °C for 2 h. The solid residue, known as holocellulose, was filtered with a nylon cloth, washed with distilled water thoroughly, and further dried in an oven at 60 °C for 16 h. Then, the holocelluloses was extracted with 10% KOH at 25 °C for 16 h with a solid to liquid ratio of 1:25 g/L. The hemicellulosic fraction (Ha) was precipitated by neutralizing the filtrate with HOAc to pH 5.5 to 6.0. Next, the filtrate was concentrated under reduced pressure, and pure ethanol was added to the filtrate with continuous stirring to a final concentration of 10% (*v*/*v*). The precipitated hemicellulosic polymer was recovered by centrifugation and marked as H_10_. The supernatant was further sequentially fractionated by graded precipitations in ethanol concentrations of 20%, 35%, 50%, 65%, and 80%, leading to hemicellulosic fractions H_20_, H_35_, H_50_, H_65_, and H_80_, respectively. All the hemicellulosic fractions were dialyzed against distilled water and then freeze-dried. Three replicates were carried out for each procedure. The scheme for the isolation and precipitation of hemicelluloses from sweet maize stems used in this work is shown in Figure 1.

### 2.3. Structural Characterization

The neutral sugars and uronic acids of the eight hemicellulosic fractions were examined by high-performance anion exchange chromatography (HPAEC) (Dionex, Sunnyvale, CA, USA). The hemicelluloses (5 mg) were hydrolyzed by 4 mL of 6% sulphuric acid at 105 °C for 2.5 h, and then the hydrolysate was diluted 50-fold with ultrapure water and injected into an HPAEC system. The molecular weights and molecular weight distributions were determined by gel permeation chromatograph (GPC) (Dionex, Sunnyvale, CA, USA) according to previous work [16].

The FT-IR spectra of the hemicellulosic fractions were recorded on a Bruker spectrophotometer (Bruker BioSpin AG, Fällanden, Switzerland) in the range of 4000 to 400 cm^−1^ with a resolution of 4 cm^−1^. A KBr disc containing 1% finely ground hemicelluloses was used for measurement. The solution–state ^1^H and ^13^C NMR spectra were recorded on a Bruker AV III 400 MHz spectrometer operating in the FT mode at 100.6 MHz. The purified hemicelluloses (15 mg for ^1^H NMR and 80 mg for ^13^C NMR) were dissolved in 1 mL of D_2_O, and then the resonance spectra were obtained. For ^1^H NMR spectra, the acquisition and relaxation times were 3.9 and 1.0 s, respectively. For ^13^C NMR spectra, the spectra were recorded at 25 °C after 30,000 scans. A 30° pulse flipping angle, 9.2 μs pulse width, 1.36 s acquisition time, and 1.89 s relaxation delay time were used. The spectral widths were 2200 and 15,400 Hz for the ^1^H and ^13^C dimensions, respectively.

## 3. Results and Discussion

### 3.1. Yield and Chemical Composition

The yield and composition of hemicelluloses varied depending on the isolation and precipitation methods. The water-soluble hemicellulosic fraction H_H2O_ was precipitated by 95% ethanol with a yield of 3.3% of the dewaxed samples, corresponding to 11.8% of the original hemicelluloses. Most hemicelluloses are intermeshed and bonded through covalent cross-linkages with lignin [17]. The fractionation of hemicelluloses from the cell wall is strongly restricted by the lignin network and lignin-carbohydrate complexes. Therefore, the sweet maize stems were delignified with acidified sodium chlorite solution and then extracted with 10% KOH to recover most of the hemicelluloses. After neutralization of the KOH-soluble liquid and concentration, Ha was precipitated from solution with a yield of 8.3% of the dewaxed samples. Then the solution was further precipitated by 10%, 20%, 35%, 50%, 65%, and 80% ethanol, resulting in six hemicellulosic fractions labeled as H_10_, H_20_, H_35_, H_50_, H_65_, and H_80_, respectively. Result showed that the yields of H_10_, H_20_, H_35_, H_50_, H_65_, and H_80_ were 3.1, 1.4%, 0.4%, 2.3%, 1.3%, and 0.4% of the dewaxed stems, corresponding to 11.1%, 5.0%, 1.4%, 8.2%, 4.6%, and 1.4% of the original hemicelluloses, respectively. The result showed that most hemicelluloses were extracted with alkali solution from the delignified material since hydroxyl ions caused swelling of cellulose, disruption of intermolecular hydrogen bonds between hemicelluloses and cellulose, and hydrolysis of ester bonds [18,19]. A total yield of 73.2% of the original hemicelluloses was fractionated from the sweet maize stems. Obviously, most hemicelluloses were obtained by KOH extraction, while fewer hemicelluloses were obtained by hot water extraction, suggesting that the alkali treatment was an effective method for the fractionation of hemicelluloses from sweet maize stems.

The contents of neutral sugars and uronic acids in these isolated hemicelluloses are shown in Table 1. As can be seen, H_H2O_ contained an abundance of glucose (27.83%), xylose (27.32%), galactose (16.81%), mannose (13.94%), and arabinose (12.65%) as well as small amounts of rhamnose (1.72%). The high content of glucose was ascribed to the existence of starch and/or α-glucan in the plant cell walls of sweet maize stems. However, in all the alkali-soluble hemicellulosic fractions, xylose was the dominant sugar, and arabinose appeared as the second major sugar. Glucose, galactose, rhamnose, mannose, and uronic acids were observed in these hemicellulosic fractions in minor amounts. The result suggested that the hemicelluloses isolated from the alkali solution were mainly arabinoxylans. Interestingly, the content of xylose in the hemicellulosic fractions H_10_ to H_80_ gradually decreased from 88.62% to 69.73% as the concentration of ethanol increased from 10% to 80%, while the content of arabinose gradually increased from 5.41% to 16.20%. Furthermore, the ratio of xylose to arabinose decreased from 16.43 to 4.21, indicating that the increase in ethanol concentrations led to the precipitation of less linear hemicelluloses. That is, hemicelluloses rich in backbone structure could be precipitated at low ethanol concentrations, while with increasing ethanol concentrations, hemicelluloses with more side chains and complex structures were obtained.

### 3.2. Molecular Weight 

To investigate the degree of degradation of these hemicelluloses during extraction, the molecular weights of all the hemicellulosic fractions were determined. Table 2 shows their weight average (*M_w_*) and the number of average molecular weights as well as polydispersity (*M_w_*/*M_n_*). It was found that the *M_w_* value was 40,430 g/mol for H_H2O_ and 43,490 to 110,400 g/mol for H_a_ and H_10_ through H_80_, suggesting that the hemicelluloses extracted with hot water had a lower *M_w_* than those extracted with alkali solution from the delignified material. This phenomenon indicated that the hot water treatment only dissolved low molecular hemicelluloses (i.e., galactoarabinoxylans, pectic substances, and *α*-glucan) [20], while the alkali treatment of holocellulose from sweet maize stems released highly molecular hemicelluloses; the *M_w_* values of hemicellulosic polymers varied depending on the precipitating ethanol concentrations. In all the alkali-soluble hemicelluloses, Ha precipitated by acidification had a low *M_w_* as compared with the hemicelluloses precipitated by ethanol. An increase in ethanol concentration from 10% to 65% led to the rise in the *M_w_* value from 61,170 to 110,400 g/mol, whereas a further increase in ethanol concentration to 80% resulted in a decrease in *M_w_* to 73,690 g/mol. The result indicated that the ethanol concentrations of 50% and 65% favored the precipitation of the hemicelluloses with higher molecular weights, whereas, with the further increase of the ethanol concentration, the molecular weight of the precipitated hemicelluloses decreased. Furthermore, it was found that as compared with the water-soluble hemicelluloses (*M_w_*/*M_n_* = 3.2), the alkali-soluble hemicelluloses had relatively lower polydispersities (*M_w_*/*M_n_* = 2.0 to 3.0). It is probable that the alkali-soluble hemicelluloses had a homogenous structure, mainly xylan-based hemicelluloses, while the water-soluble hemicellulosic polymer was a mixture of hemicelluloses, pectic substances, and *α*-glucan as revealed by sugar analysis.

### 3.3. FT-IR Spectra Analysis 

**Figure 2:**
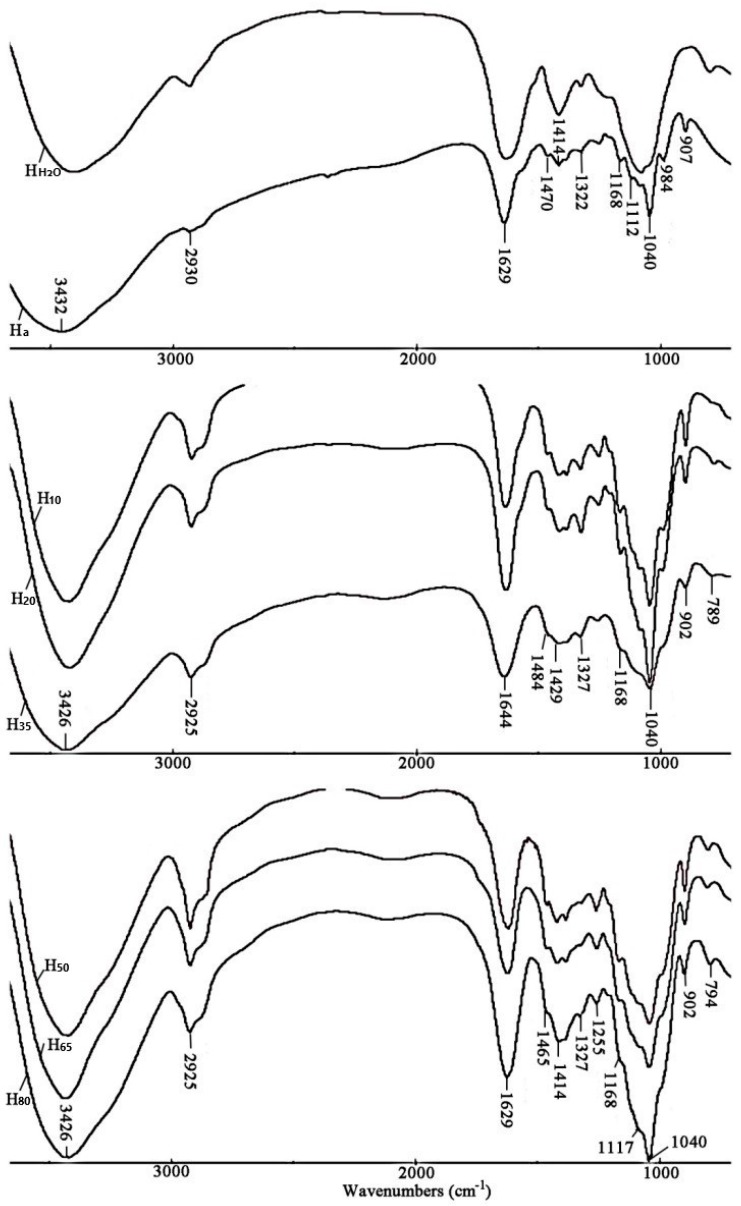
FT-IR spectra of the hemicellulosic fractions.

The FT-IR spectra of all the hemicellulosic fractions obtained are shown in Figure 2. No significant difference in the main absorptions was observed among the alkali-soluble hemicelluloses, suggesting a similar chemical structure of the hemicellulosic polymers. The bands at 1465, 1414, 1385, 1327, 1255, 1168, 1040, 984, and 902 cm^−1^ are characteristic peaks of hemicelluloses [21,22]. A predominant absorption at 1040 cm^−1^ is due to the C–O–C stretching of glycosidic linkages of xylans [9,23]. The presence of arabinose as side chains on xylans is indicated by the absorption at 1168 cm^−1^ [24]. A low intensity of the signal at 984 cm^−1^ also indicated the presence of arabinose units, and it has been reported that arabinose was attached at the C–3 position of the xylopyranosyl constituents [24]. A characteristic band at 902 cm^−1^, corresponding to the C1 group frequency or ring frequency, is assigned to the *β*–(1, 4)–glycosidic linkages in the hemicellulosic polymers [25]. This band was observed in all the alkali-soluble hemicelluloses but not observed in the water-soluble hemicelluloses, which was probably due to the fact that the relative content of xylose in the alkali-soluble hemicelluloses was remarkably higher than that in H_H2O_. In addition to the bands at 1168, 1040, 984, and 902 cm^−1^, the absorptions at 1465 and 1414 cm^−1^ represent symmetric stretching vibrations of glucuronic acid groups and those at 1385 and 1255, 1327 cm^−1^ represent C–H stretching and O–H or C–O bending vibrations.

### 3.4. ^1^H and ^13^C NMR Spectra Analysis 

**Figure 3:**
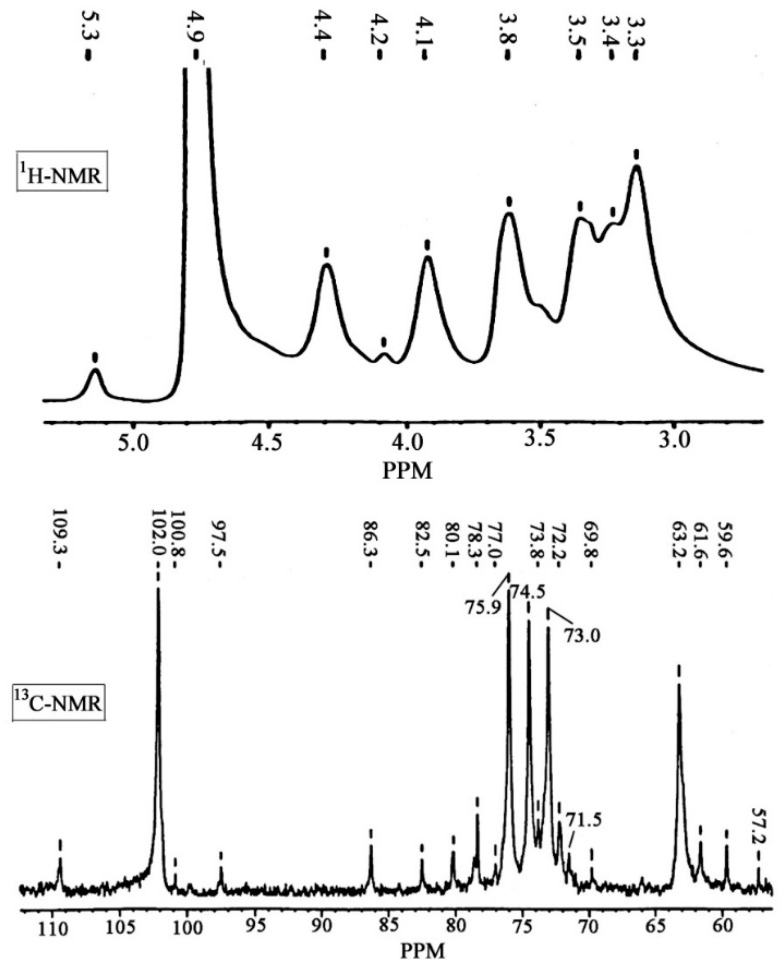
^1^H and ^13^C NMR spectra of the hemicelluloses H_65_ obtained from sweet maize stems.

To further elucidate the structural information of the hemicellulosic polymers, the hemicellulosic fractions H_65_ were investigated by using the ^1^H and ^13^C NMR spectra (Figure 3). For ^1^H NMR spectra, the relevant signals occurred in two regions: the anomeric region (5.60 to 4.90 ppm for *α* anomers and 4.90 to 4.30 ppm for *β* anomers) and the ring proton region (4.50 to 3.00 ppm) [26,27]. It was found that the intensity of *β* anomers was higher than that of *α* anomers, confirming that the xylose units were linked *β*–glycosidically, which was in accordance with the presence of absorption at 902 cm^−1^ in the FT-IR spectra. The six main signals at 4.4 (H–1), 4.1 (H–5eq), 3.8 (H–4), 3.5 (H–3), 3.4 (H–5ax), and 3.3 (H–2) ppm are assigned to non-substituted backbone of *β*–d–xylopyranosyl units [23]. Additionally, a small signal at 5.3 ppm was related to anomeric protons of terminal *α*–d–arabinofuranosyl. For ^13^C NMR spectra, *β*–(1, 4)–linked xylopyranosyl units are characterized by the five main signals at 102.0, 75.9, 74.5, 73.0, and 63.2 ppm, which corresponded to the C–1, C–4, C–3, C–2, and C–5 positions, respectively [6]. The signals at 109.3, 86.3, 80.1, 78.3, and 61.6 ppm are indicative of C–1, C–4, C–2, C–3, and C–5 of *α*–l–arabinofuranosyl units linked to *β*–d–xylans, respectively. Moreover, some small signals at 97.5, 82.5, 72.2, and 59.6 ppm corresponding to C–1, C–4, C–5, and OCH_3_ of the 4–*O*-methyl–d–glucoronic acids, respectively, were also observed [28]. Therefore, based on the results of sugar analysis and FT-IR and NMR spectra it could be concluded that the alkali-soluble hemicelluloses extracted from the holocellulose of sweet maize stems were mainly composed of arabinoxylans.

## 4. Conclusions

The hemicellulosic polymer from sweet maize stems was firstly fractionated with water and alkaline treatment and then fully characterized by wet chemical and spectral technologies. The water-soluble hemicelluloses were rich in glucose, xylose, and galactose with a relatively low molecular weight. In comparison, the alkali-soluble hemicelluloses were rich in xylose and arabinose with high molecular weight and polydispersity. Although the core of hemicellulosic biomacromolecule could be maintained and isolated under alkaline conditions, the polymer with less branches was easier to be precipitated at a low ethanol concentration. The hemicellulosic fraction with a more complex structure could be isolated with increasing ethanol concentration. The effective strategy for the fractionation and basic understanding of hemicelluloses hopefully provides new opportunities for future applications.

## Figures and Tables

**Table 1:** Contents of neutral sugars and uronic acids (relative %, *W*/*W*) in the isolated hemicellulosic fractions.

Sugars (%)	H_H_2_O_	H_a_	H_10_	H_20_	H_35_	H_50_	H_65_	H_80_
Rhamnose	1.72	ND	ND	ND	ND	0.25	ND	0.11
Arabinose	12.65	4.21	5.41	5.57	6.11	7.65	9.11	16.20
Galactose	16.81	ND	0.42	0.86	1.21	0.87	1.56	4.18
Glucose	27.83	0.39	0.94	1.04	2.13	1.67	3.55	3.13
Mannose	13.94	ND	ND	ND	ND	ND	ND	0.25
Xylose	27.32	90.10	88.62	85.73	83.22	82.14	80.23	69.73
Uronic acids	ND ^a^	5.30	4.70	6.80	7.33	7.67	5.55	6.49
Xyl/Ara ^b^	2.17	21.40	16.43	15.39	13.62	10.74	8.81	4.21

^a^ ND, not detectable. ^b^ Represent xylose to arabinose ratio.

**Table 2:** Weight-average (*M_w_*) and number-average (*M_n_*) molecular weights and polydispersity (*M_w_*/*M_n_*) of the hemicellulosic fractions isolated from sweet maize stems.

	H_H_2_O_	H_a_	H_10_	H_20_	H_35_	H_50_	H_65_	H_80_
*M_w_*	40,430	43,490	61,170	72,940	76,040	99,520	110,400	73,690
*M_n_*	12,630	18,910	26,360	24,310	34,420	36,860	55,200	35,090
*M_w_*/*M_n_*	3.2	2.3	2.7	3.0	2.5	2.7	2.0	2.1
